# Large-Scale Metasurface Simulation Using Local-Segmented Approach

**DOI:** 10.3390/ma18030649

**Published:** 2025-01-31

**Authors:** Shiyao Wang, Site Zhang, Naitao Song, Donglin Xue

**Affiliations:** 1 Changchun Institute of Optics, Fine Mechanics and Physics, Chinese Academy of Sciences, Changchun 130033, Chinasongnaitao@ciomp.ac.cn (N.S.); 2 University of Chinese Academy of Sciences, Beijing 100049, China; 3 State Key Laboratory of Advanced Manufacturing for Optical Systems, Chinese Academy of Sciences, Changchun 130033, China

**Keywords:** large-scale metasurface simulation, field decomposition, segmented computation, domain decomposition, rigorous-coupled wave analysis, Fourier modal method

## Abstract

The complicated electromagnetic couplings between nanostructures present substantial challenges in the design and simulation of metasurfaces, especially large-scale elements. The couplings are typically neglected in a conventional simulation. We introduce a computational framework that includes the electromagnetic coupling effects between meta-atoms. Decomposing the incident field and segmenting the computing range for individual local simulations allows for an effective and accurate simulation of the entire metasurface. Numerical examples of a 2 mm diameter cylindrical metalens with a numerical aperture of 0.9 and a 1 mm aperiodic beam splitter show the deviation from the conventional method is reduced by 97% compared to the rigorous method, while the computation times are 10 times and 4 times faster than the rigorous methods, respectively.

## 1. Introduction

Vector electromagnetic field simulation technology is a powerful method for designing and simulating micro- and nano-optical elements, such as optical metasurfaces, primarily focusing on how to efficiently simulate large-scale metasurfaces with higher accuracy [1,2]. Rigorous simulation methods, e.g., the finite-difference-time-domain (FDTD) method [3], are only feasible for small elements. Simulation of entire large-scale metasurfaces, ranging from hundreds of micrometers to several centimeters, is extremely challenging due to the unacceptable physical memory and computing time requirements.

The conventional strategy to rapidly model a large-scale metasurface is the local periodic approximation (LPA) method [4]. It relies on the assumption that each meta-atom in the metasurface is placed on a periodic arrangement. LPA has been proven to work well for many metasurface designs [4,5,6], but it is unsuitable for designs requiring large phase gradients, such as high numerical aperture (NA) lenses [2,7,8,9]. The problem arises from the electromagnetic coupling effects between adjacent meta-atoms, which are not accounted for in LPA. To alleviate this issue, domain decomposition methods divide the entire element into a sequence of sub-domains with different simulation methods and boundary conditions, e.g., simulated by FDTD with perfectly matched layers (PML) [10] for overlapping [11] and non-overlapping sub-domains [12], the aperiodic Fourier modal method (FMM, also named rigorous coupled wave analysis) for non-overlapping sub-domains [13], and the finite element method (FEM) with a periodic boundary condition for non-overlapping sub-domains [14]. Although coupling effects within sub-domains are incorporated and benefit the numerical efficiency, the PML boundary condition limits the design of elements by necessitating minimal coupling effects between sub-domains [15], and the periodic boundary condition asks for the periodicity of the entire element [14]. S. Linss et al. proposed an adapted finite-difference beam propagation method (FD-BPM) to decrease the numerical effort [16]. However, as a semi-rigorous simulation method, BPM does not precisely model evanescent modes, which leads to omitting some resonator-like effects. Therefore, a computational solution that includes electromagnetic coupling and good numerical efficiency is strongly needed for large-scale metasurface simulation [17,18].

In this article, we propose a segmented approach for simulating large-scale metasurfaces. Our approach introduces field decomposition to previous domain decomposition methods. The decomposed computing ranges are defined by electromagnetic coupling effects excited from the decomposed fields. By adjusting the size of the decomposed fields and the overlapping computing range, our efficient approach includes the electromagnetic field coupling effects between adjacent meta-atoms in the simulations. Moreover, the numerical effort is linear to the number of segments, which benefits the effectiveness of metasurface simulation, particularly for large-scale elements. As a proof of concept, numerical examples of a 2 mm diameter cylindrical metalens with NA=0.9 and a 1 mm diameter aperiodic beam splitter were designed and simulated. Compared with rigorous methods and LPA, the advantages of our approach are discussed in terms of the effectiveness and accuracy.

## 2. Theory of Local-Segmented Approach

Beginning from the localized electromagnetic coupling effects between adjacent meta-atoms, we propose a local-segmented approach (LSA) to combine the decomposed incident fields and computing range into sub-segments. This approach, transforming the global simulation of an entire element into the independent simulation of sub-fields and sub-computing ranges has an advantage in computing efficiency, while reasonably considering the electromagnetic coupling effects.

### 2.1. Transverse Localized Electromagnetic Coupling Effects

**Figure 1:**
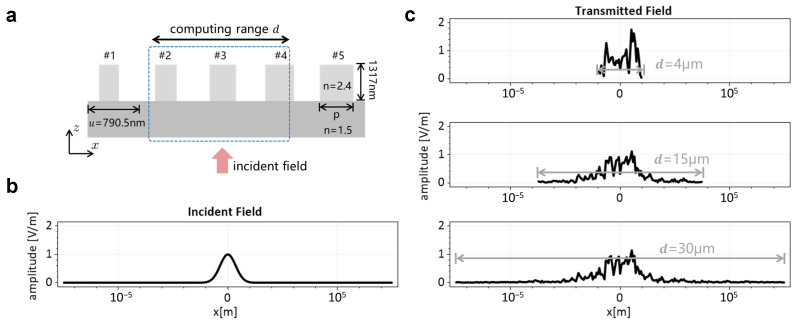
(**a**) Schematic of the blazed-metagrating in a grating period; (**b**) amplitude distribution of a fundamental Gaussian beam, with 1µm beam waist; (**c**) Amplitude distribution of the transmitted fields of the metagrating normal illustrated by fundamental Gaussian beam. Computing range *d* is 4µm (**top**), 15µm (**middle**), and 30µm (**bottom**), respectively.

We designed a one-dimensional (1D) metagrating, which relies on the library of 1D meta-atoms at wavelength λ=1550nm [19]: TiO_2_ pillars on top of a silicon dioxide substrate, as shown in Figure 1a. One-dimensional means the permittivity and permeability are invariant along the *y*-axis. The height of the pillar is fixed to 1317nm, and the period *u* is 790.5nm. The pillar width varies from 80nm to 700nm (imposing a minimum feature size of 1nm), forming a 2π phase profile library for a TM-polarized field. The library of meta-atoms is used throughout this article. The grating period $\Lambda_{x}$ along the *x*-axis includes five meta-atoms, of which the pillar widths are {158,316,395,474,632} nm, respectively. Considering a fundamental Gaussian beam with a 1 µm beam waist (as shown in Figure 1b), normal incident upon the element, the transmitted (or reflected) field could be simulated by rigorous methods, e.g., the Fourier modal method (FMM, also named rigorous coupled wave analysis, RCWA) [20,21]. Maintaining the incident field unchanged, we alter the size of the transverse computing range *d*. What we need to emphasize is that the *d* is chosen arbitrarily, it is not just a periodic structure. As shown in Figure 1c, the amplitude in the central region of the transmission fields becomes gradually stable within d=15µm, because the metasurfaces typically do not involve long-range electromagnetic coupling propagating along the transverse direction. Therefore, if a narrow beam is incident upon a metasurface, we can define a transverse computational range *d* centered on the point of incidence (the size of *d* is usually larger than the transverse width of a narrow incident field). The electromagnetic coupling effects within this range will be simulated, while the weak effects beyond the range *d* will be ignored. Considering LPA and other domain-decomposition methods, the incident fields within a decomposed domain are the same size as the computing range, and the coupling effects are truncated prematurely, which leads to less accurate simulation results for applications involving adjacent meta-atoms with strong coupling effects.

By decomposing the incident field and computing range, the local transverse electromagnetic effects are included in the simulation, which is typically suitable for general metasurfaces since they are always used in the far-field and will not excite transverse propagation modes (or guided modes). There will be other applications, such as the excitation of surface resonances [22], in which the long-range transverse propagation is crucial, but such propagation implies a nearly periodic boundary condition in which the LPA will be accurate. In Section 3.1, we prove that the LPA can be regarded as a boundary scenario of our approach; these two approaches are complementary.

### 2.2. Local Segment Approach

**Figure 2:**
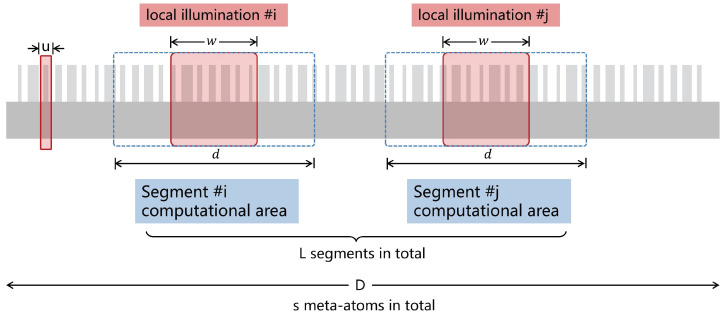
Schematic diagram of local segmented approach. An arbitrary metasurface (consisting of meta-atoms with period *u*) is decomposed into overlapped sub-segments. Each segment is illustrated by a local indent field with a width of *w* and a computing range with the size of *d*.

With the transverse local coupling effects, we propose a local segment approach (LSA) for large-scale metasurface simulation. Figure 2 illustrates the schematic of the decomposition of an arbitrary incident field and an aperiodic metasurface into sub-segments. For each decomposed field, computing the electromagnetic field within its local effect response range *d* reduces the computing range of each sub-segment, while accurately simulating the coupling effect between adjacent meta-atoms, thereby reducing the overall computing complexity.

**Figure 3:**
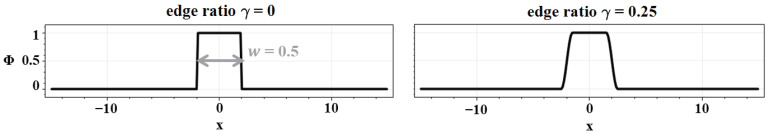
Distribution of window function Φ(x) with w=0.5, γ=0 (**left**), and γ=0.25 (**right**).

Following the work of Asoubar et al. [23], an arbitrary incident field $E^{\mathrm{in}}\left(x\right)$ could be mathematically decomposed to a series of local fields $\left\{E_{i}^{\mathrm{in}}\left|i=1,\cdots L\right.\right\}$ by

(1) $$\begin{array}{cc} \; & E^{\mathrm{in}}\left(x\right)=\sum_{i=1}^{L}E_{i}^{\mathrm{in}}\left(x\right)\\ \; & E_{i}^{\mathrm{in}}\left(x\right)=\Phi_{i}\left(x\right)E^{\mathrm{in}}\left(x\right) \end{array},$$

where Φ is a window function with local carrier, and $\Phi_{i}\left(x\right):=\Phi \left(x-x_{i}\right)$ with the supporting points $x_{i}$. The property

(2) $$\sum_{i=1}^{L}\Phi_{i}\left(x\right)=1$$

should be maintained for all points *x* within the defined area of the incident field $E^{\mathrm{in}}\left(x\right)$. The window function is defined as

(3) $$\Phi \left(x\right)=\left\{\begin{array}{cc} 1\; & \mathrm{for}\;|x|⩽\frac{w}{2}\left(1-\gamma \right),\\ 0\; & \mathrm{for}\;|x|⩾\frac{w}{2}\left(1+\gamma \right),\\ 0.5\left[\cos\left(\frac{\pi }{w\gamma }\left(x-\frac{w}{2}\left(1-\gamma \right)\right)\right)+1\right]\; & \mathrm{else}. \end{array}\right.$$

where *w* is the width of a local field, and γ is the ratio of smooth edge. w,γ∈R, w>0, and 0⩽γ⩽1. The distribution of the window function is shown in Figure 3.

The Fourier transform of a local field $E_{i}^{\mathrm{in}}\left(x\right)$ is

(4) $${\tilde{E}}_{i}\left(k_{x}\right)=F\left[E_{i}^{\mathrm{in}}\left(x\right)\right]\left(k_{x}\right),$$

where $k_{x}$ is the spatial frequency. By selecting a computing range with size d⩾w, a segmented metastructure *i* with the permittivity

(5) $$\epsilon_{i}\left(x\right)=\left\{\begin{array}{cc} \epsilon (x) & |x-x_{i}|⩽\frac{d}{2},\\ 0 & \mathrm{else} \end{array}\right.$$

is defined (same for its permeability μ(x)). By expanding into Fourier modes and solving the eigenfunctions with FMM, its bidirectional propagating scattering matrix S can be calculated by boundary conditions as in Ref. [24]. The corresponding output field is

(6) $${\tilde{E}}_{i}^{\mathrm{out}}\left(k_{x}^{\prime }\right)=S_{i}^{d}\left[{\tilde{E}}_{i}^{\mathrm{in}}\left(k_{x}\right)\right]\left(k_{x}^{\prime }\right),$$

with computing size *d* for the segment *i*. Each local field with a width of *w* and the computing range of size *d* constitute a segment, which may overlap or stitch with the adjacent segments depending on the numerical parameters *w*, γ, and *d*.

The coherent superposition of all the output fields, the entire output field ${\tilde{E}}^{\mathrm{out}}$ in the spatial frequency domain, is obtained as

(7) $${\tilde{E}}^{\mathrm{out}}\left(k_{x}\right)=\sum_{i=1}^{L}{\tilde{E}}_{i}^{\mathrm{out}}\left(k_{x}\right).$$

Then, the output field in spatial space could be calculated by the inverse Fourier transform

(8) $$E^{\mathrm{out}}\left(x\right)=F^{-1}\left[{\tilde{E}}^{\mathrm{out}}\left(k_{x}\right)\right]\left(x\right).$$

The process from Equation (1) to (8) constitutes a complete local-segmented approach for modeling the output field of a metasurface. Utilizing field and computing range decomposition, the electromagnetic coupling effects excited by each local illumination are simulated locally and individually. The size of each sub-segment is much smaller than the entire metasurface. In this case, the computing complexity of this method is linearly scaled to the number of sub-segments, which significantly improves the computational efficiency compared to rigorous simulations.

### 2.3. Parameters of Local Segments

The simulation accuracy depends on the parameters w,d, and γ in Equations (3) and (6). By simulating a local field decomposed from TM-polarized plane wave normal incident upon the metagrating in Section 2.1, we discuss influences under the variation of these parameters. The local field width *w* varies from 0.5 to 5µm, with the step of 0.5µm. The smooth edge ratio γ varies from 0 to 0.25, with the step of 0.05. The computing size *d* varies from 1 to 30µm, with the step of 1µm. Utilizing the simulation results of $d_{\mathrm{ref}}=\Lambda_{x},\;\gamma_{\mathrm{ref}}=\gamma$ as reference fields, the deviation between *E* and $E^{\mathrm{ref}}$

(9) $$\sigma \left(E,E^{\mathrm{ref}}\right):={\left(\frac{\sum_{x}{\left|E\left(x\right)-E^{\mathrm{ref}}\left(x\right)\right|}^{2}}{\sum_{x}{\left|E^{\mathrm{ref}}\left(x\right)\right|}^{2}}\right)}^{1/2}.$$

**Figure 4:**
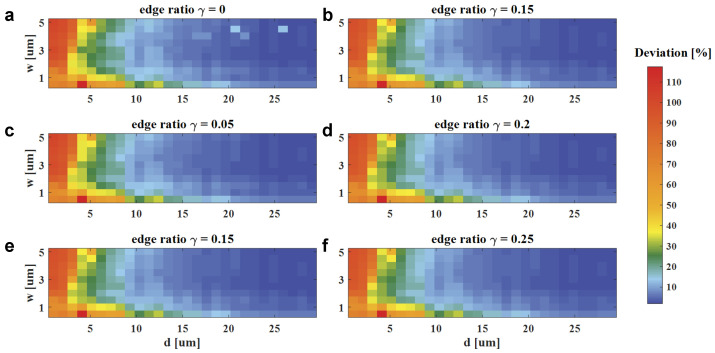
Deviation distributions of the local simulated fields with respect to reference fields at different modeling parameters. (**a**)–(**f**) With same horizontal and vertical coordinates, local field width *w* varies from 0.5 to 5µm; computing size *d* varies from 0.5 to 30µm. Smooth edge ratio γ varies from 0 to 0.25 for each sub-figure, respectively.

The deviation distributions at different modeling parameters are shown in Figure 4. To quantitatively describe the convergence of deviation, we define that, as d increases, the results are considered converged when five consecutive deviation values are all less than 3% (other thresholds can be selected based on the required accuracy). The minimum *d* for a convergent deviation is summarized in Table 1. It demonstrates the following:

- If *w* and γ are constant, the deviation tends to convergence as the computing size *d* increases, which is because more electromagnetic coupling effects are included in the segments for a larger *d*;
- Comparing Figure 4a with Figure 4b–f, if *w* and *d* are constant, the deviation is more likely to tend towards convergence when the edge ratio γ is a non-zero value. In contrast to other domain decomposition methods to decouple the coupling effects between sub-domains, the coupling effects are included by a decomposed field with the non-zero edge ratio γ;
- As for local field width *w*, a relatively small *w* (w<u) will generate strong transverse effects, which leads to a larger computing size for deviation convergence. Conversely, a larger *w* takes a longer computing size *d* to converge. Therefore, a proper *w* is usually chosen as several times of periods *u* to the meta-atoms.

The selection of these parameters is flexible and adjustable. Once *w* and γ have been selected, opting for a smaller *d* can enable conducting a rapid preliminary evaluation during the initial phases of metasurface design. For additional validation and fabrication processes, a larger *d* captures more electromagnetic field coupling effects, while achieving higher computing efficiency than rigorous methods.

## 3. Numerical Examples

To clarify the algorithm we propose with respect to rigorous methods and the conventional approximated method, we define:

- Global: rigorous methods simulated by FMM of the entire element;
- LSA: local-segmented simulation defined in Equations (1)–(8);
- LPA: fields of an aperiodic element are approximated by individually simulating each meta-atom with periodic boundary conditions as [4]. The amplitude is considered despite the minimal impact.

Those methods were applied in selected cases and compared in terms of the modeling accuracy and numerical efficiency. A workstation with Intel Xeon Platinum 8375C CPU (Intel Corp., Santa Clara, CA, USA, 32 cores, and 512 GB RAM) was employed in the following computing examples.

### 3.1. Numerical Complexity

Let us initially focus on the computing complexity before exploring the topic of accuracy. The global simulation of an entire element is approximately proportional to $O(N^{3})$, with *N* as the truncated order in Fourier harmonics required for the entire element of diameter *D*. For a segment with computing size d⩽D and harmonics n⩽N, its complexity is proportional to $O(n^{3})$. Assuming the incident field is decomposed into *L* local fields, the overall complexity of LSA is a linear function as $L\times O(n^{3})$, which is also a linear scaling to the entire element diameter *D*. If the computing parameters are set as w=d=D, γ=0, there is only a segment, i.e., L=1,n=N. The LSA is transformed into a boundary scenario—global simulation. The LPA can be regarded as another boundary scenario of the LSA, with w=d=u and γ=0. Its complexity is $s\times O(n_{u}^{3})$, where *s* denotes the number of meta-atoms in the metasurfaces, and $n_{u}$ is the number of harmonics required for computing size *u*. Therefore, LSA is a scalable simulation method that allows for adjustable precision and efficiency, acting as a unified theoretical framework that bridges the global method and LPA.

**Figure 5:**
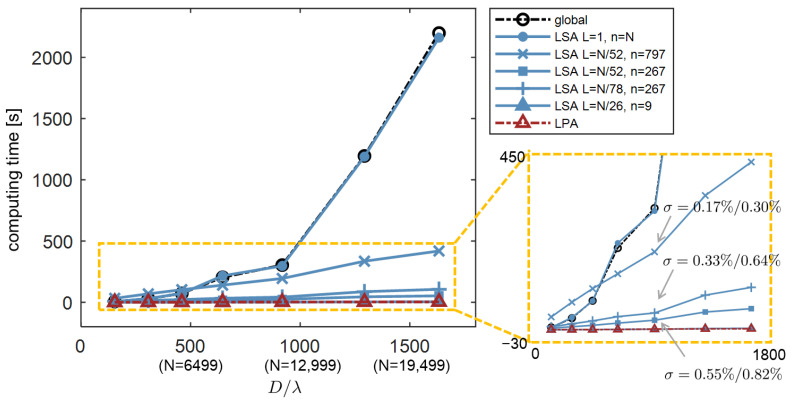
Computing time versus diameter of metasurfaces that are simulated using different approaches: Global, LSA, and LPA, respectively. Three deviations σ of the simulated fields (without evanescent modes / with evanescent modes) between LSA and rigorous methods are given.

The complexity of the three approaches is summarized in Table 2. We performed simulations for the metagrating given in Figure 1a using the three different methods. The time required to simulate the entire element as a function of its diameter *D* is plotted in Figure 5. Except for the boundary scenario of L=1 and n=N, the computing time of the LSA is almost linearly proportional to the entire diameter *D*. As for the two boundary scenarios of LSA, they show good agreement in computing time with the Global approach and LPA at the same simulated accuracy, respectively. The figure also shows that the overall computing time of LSA is decided by the local field width *w* (w=D/L) and the computing size *d* (which corresponds to the harmonics *n*). A slightly larger local field width *w* and smaller computing size *d* results in higher computing efficiency. We compare the simulation results with a global method at three specific simulations for fields without evanescent modes and with evanescent modes, as the deviation given in Figure 5.

### 3.2. Simulation of a Metalens

**Figure 6:**
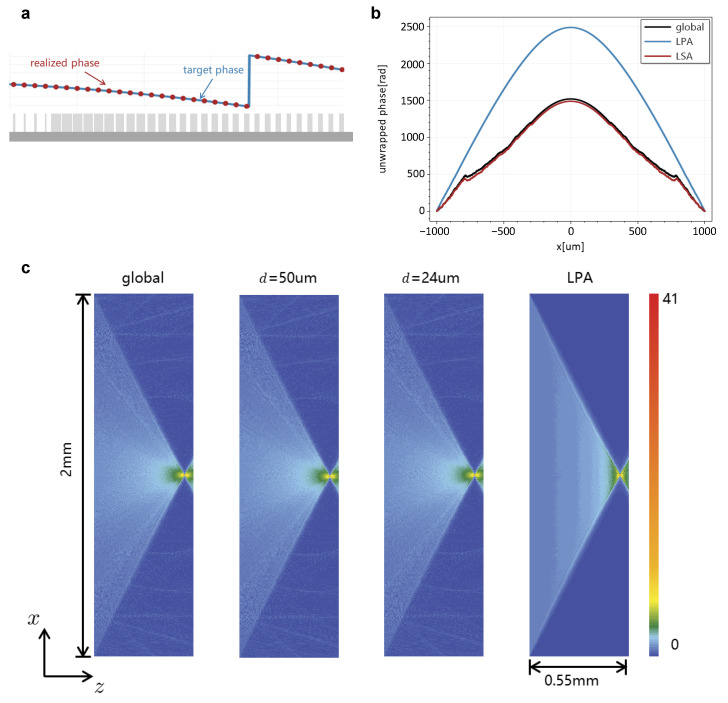
(**a**) Schematic of cylindrical lens design. (**b**) The unwrapped phase profile of transmitted field to the cylindrical lens, simulated by Global, LSA, and LPA approaches, respectively. (**c**) Amplitude distribution of the $E_{y}$ field component in the *x*–*z* plane, simulated by Global approach, LSA with d=24µm, LSA with d=50µm, and LPA, respectively.

We designed a 2 mm diameter cylindrical metalens with focal length f=0.5 mm. The NA of the metalens was 0.90, which is rather challenging for a conventional simulation, because the strong phase divergence violates the periodic approximation of LPA. According to the schematic shown in Figure 6a, the phase modulation for a normal incidence field is designed by

(10) $$\Delta \psi \left(x\right)=-k_{0}n_{\mathrm{air}}\left(\sqrt{x^{2}+f^{2}}-f\right),$$

where $k_{0}=2\pi /\lambda$, with λ as the designed wavelength. $n_{\mathrm{air}}$ is the refractive index of air. The metalens is structured by the phase-pillar library provided in Section 2.1 under the LPA method.

A TM-polarized plane wave with the amplitude of one normal was illustrated on the metalens, and the transmitted fields immediately behind the metalens were simulated by the three methods. The unwrapped phase profile is shown in Figure 6b. Although the unwrapped phase of LPA faithfully replicates the desired phase profile as in Equation (10), it is quite distant from that of the Global approach. It is worth mentioning that the simulation by LPA indicates that the design does manage to produce a desired phase profile that works well under the assumption of LPA. However, the design contains rapid structural variations that lead to strong coupling effects that violate the periodic assumption, making this element unsuitable for LPA simulation. The local incident field width in the LSA simulation was set as w=4µm, with smooth edge ratio γ=0.25. Two simulations with computing size d=50µm and 24µm were performed, respectively. The difference between the LSA and the Global method obviously decreased compared with that of LPA since we introduced coupling effects, and it continued to decrease with the increase in the computing size *d*. As shown in Table 3, the deviation of d=24µm compared to the rigorous method was reduced by 93% (for the propagating fields and 91% for fields with evanescent modes) relative to the LPA. It continued decreasing for the simulation at a larger computing size d=50µm (97% for both fields with and without evanescent modes) with an acceptable increase in the computing time (Decrease from 10 times to 4 times faster than the Global method). The LSA performed much better in terms of the agreement with rigorous simulations than the LPA in this example.

The spectrum plane wave (SPW) [25] method was been used to simulate the field propagation along the *x*-*z* plane, as shown in Figure 6c. It shows good agreement between the Global and LSA, even for the scattering caused by coupling between adjacent meta-atoms, which is omitted and invisible in the simulation results of LPA. That also leads to the difference in focusing efficiency between the LPA and Global methods, as shown in Table 3. The focusing efficiency is defined as the ratio of total electric field power in the aperture within the Airy disk to the total electric field power of incident light,

(11) $$\eta_{\mathrm{focus}}=\frac{\int_{\mathrm{Airy}}\langle S^{\mathrm{out}}\rangle \cdot dA}{P^{\mathrm{in}}},$$

where $P^{\mathrm{in}}$ is the power of the incident beam. 〈S〉 stands for the time-average pointing vector. A is the surface vector.

The performance of the simulation shows that the LSA is accurate even for the metalens with a large NA of 0.9. The computing time of the three approaches is listed in Table 3. The LSA is 9.5 times faster than the Global method in this example with a 1% deviation; it can be much quicker (about 31 times) without losing much accuracy (4% deviation). The computing time can be even faster if a larger local incident width *w* and a smaller computing size *d* are determined. As discussed in Section 3.1, the time advantage becomes more pronounced for a larger scale of metasurfaces. Therefore, a rigorous simulation of large NA and large-scale metalenses becomes possible. We also expect that LSA will become even more critical in metasurfaces with structures that exhibit strong electromagnetic coupling between adjacent meta-atoms.

### 3.3. Simulation of a Beam Splitter

**Figure 7:**
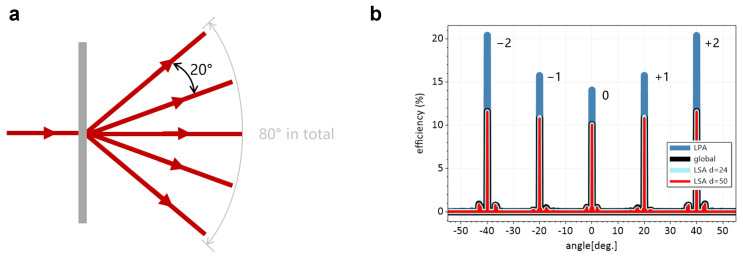
(**a**) Schematic of metasurface beam splitter. A normal incident beam is split into five beams with uniform power distribution. Angle between two adjacent beams is 20°. (**b**) Comparison of efficiency distribution with respect to different angles simulated by Global, LSA, and LPA methods, respectively.

We designed a diameter D=1 mm beam splitter to separate the TM-polarized normal incident beam into five beams of light with uniform power distribution. The angle between two adjacent beams was 20°, as shown in Figure 7a. This beam splitter is an aperiodic element, because periodic structures can only generate modes with integer multiples of spatial frequency, which results in different angles between adjacent beams. The amplitude of the desired beams in the spatial frequency domain should be proportional to

(12) $$∥\tilde{E}∥\propto {\left(k_{0}^{2}n_{\mathrm{out}}^{2}-k_{x}^{2}\right)}^{-\frac{1}{4}},$$

where $n_{\mathrm{out}}$ is the refractive index of the output media. The desired phase profile of this target amplitude distribution was generated by the Iterative Fourier Transform Algorithm (IFTA) [26]. Designed by the same meta-atoms library as above, the total amount of meta-atoms was 1267. This beam splitter constructed by these aperiodic meta-atoms split the normal incident beam into beams with the angles of {−40.16°, −19.87°, 0°, 19.87°, 40.16°}, respectively.

As shown in Figure 7b, the transmitted efficiency distribution simulated by the LPA is different from that of the Global simulation. The efficiencies of the larger angles had more obvious differences than those of smaller angles. The LPA method overestimates the performance, since it neglects the electromagnetic coupling between adjacent meta-atoms. The local incident field for the LSA simulation was set as w=4µm, and γ=0.25. The computing size of the two simulations was d=50µm and d=24µm, respectively. As listed in Table 4, the deviation between the LSA and Global showed better agreement than that of the LPA (by a factor of 10), whether near field or far field (i.e., with or without evanescent modes). Increasing the computing size *d* also increased the modeling accuracy but with a trade-off for a slightly longer computing time. Therefore, a quick response can be conducted with a smaller *d* in the initial design process, and a larger *d* can be chosen for a more accurate simulation result for further validation. The efficiencies of the five target angles are also listed in Table 4.

The LSA showed good agreement with respect to a rigorous simulation and shorter computing time, as listed in Table 4. This example shows the advantages of our approach for an aperiodic element. Unlike periodic beam splitters and metalenses with structural symmetry, which can simplify the calculations using symmetry or periodicity [27,28], the aperiodic beam splitters require simulation methods that are more generally applicable. We expect this method will be more powerful for applications of elements without periodicity, such as beam steering over large angles and computer-generated holography (CGH).

## 4. Conclusions

We propose a local-segmented approach for the efficient and accurate simulation of large-scale nano- and micro-optical elements such as metasurfaces or CGH. This approach demonstrates good agreement with rigorous simulations, utilizing the FMM as a benchmark. By decomposing the incident field and all the elements into many segments and simulating them independently, we achieve an apparent reduction in computing time, which shows linear scaling to the number of segments. In contrast to previous domain decomposition work, field decomposition was applied, which contains benefits including the electromagnetic coupling effects between adjacent nanostructures. This approach is particularly suitable for simulating large-scale elements with strong coupling effects, where rigorous methods like the FDTD and FMM would demand too many numerical resources, while simplified approaches like the LPA may lack the requisite accuracy.

The application of a cylindrical metalens with a large NA of 0.9 was demonstrated. It is possible to simulate entire elements with our approach with much higher accuracy than the LPA (deviation from the rigorous decreased by 97%) within an acceptable computing time. The beam splitter simulation shows the applicability of our approach for applications of elements without the limitation of periodicity. The efficiency and accuracy of our approach are flexible by substituting different segmented parameters. A fast-responding simulation can be conducted during the optimization process. By adjusting the parameters, a more accurate simulation can be realized at the end of the design process for further validation. We believe this method has broad application prospects, such as imaging, holography, and beam steering in LIDAR systems. It can also meet the demands for the design and simulation process of large-scale metasurfaces.

## Figures and Tables

**Table 1:** Minimum values of *d* to obtain a convergent deviation (σ<3%) at different *w* and γ.

		w**	0.5	1	1.5	2	2.5	3	3.5	4	4.5	5
	*d*											
γ												
0			N *	N	N	N	26	27	27	N	N	27
0.05			N	N	N	N	26	26	27	27	26	27
0.1			N	N	N	N	25	26	27	26	26	27
0.15			N	N	N	N	25	24	27	26	26	26
0.2			N	N	N	N	25	25	26	26	26	25
0.25			N	N	N	N	24	25	26	26	25	25

* N means not reaching convergence. ** Units of *w* and *d* are both µm.

**Table 2:** Comparison of the numerical complexity of Global, LSA, and LPA approaches.

Approach	Segments	Number of Harmonics	Complexity
Global	1	*N*	$O(N^{3})$
LSA	*L*	*n*	$L\times O(n^{3})$
LPA	*s*	$n_{u}$	$s\times O(n_{u}^{3})$

**Table 3:** Comparison of the simulation results of metalens using Global, LSA, and LPA approaches.

Approach	Deviation (Without Evan.)	Deviation (With Evan.)	Computing Time	Focusing Efficiency
Global	-	-	1189.89s	12.52%
LSA d=50µm	1.46%	2.65%	124.51s	12.46%
LSA d=24µm	4.03%	7.38%	37.99s	12.29%
LPA	57.69%	77.71%	2.38s	21.46%

**Table 4:** Comparison of the results of beam splitter simulated by Global, LSA, and LPA approaches.

Parameters	Global	LSA d=50 µm	LSA d=24 µm	LPA
Deviation (without evan.)	-	1.33%	3.56%	45.09%
Deviation (with evan.)	-	2.09%	6.65%	66.15%
Computing time	211.64 s	57.43 s	16.65 s	0.96 s
−2 efficiency	11.59%	11.55%	11.59%	20.41%
−1 efficiency	10.83%	10.77%	10.96%	15.75%
0 efficiency	10.07%	10.05%	10.10%	14.07%
+1 efficiency	10.87%	10.81%	10.99%	15.76%
+2 efficiency	11.60%	11.56%	11.30%	20.38%
Summarized efficiency	54.96%	54.73%	55.25%	86.37%

## Data Availability

Data underlying the results presented in this paper are not publicly available at this time due to privacy reasons, but may be obtained from the authors upon reasonable request.

## References

[1] Chen W.T., Zhu A.Y., Capasso F. (2020). Flat optics with dispersion-engineered metasurfaces. Nat. Rev. Mater..

[2] Kuznetsov A.I., Brongersma M.L., Yao J., Chen M.K., Levy U., Tsai D.P., Zheludev N.I., Faraon A., Arbabi A., Yu N. (2024). Roadmap for Optical Metasurfaces. ACS Photonics.

[3] Yee K. (1966). Numerical solution of initial boundary value problems involving maxwell’s equations in isotropic media. IEEE Trans. Antennas Propag..

[4] Robben B., Penninck L. (2024). Simulation methods for large-area meta-surfaces: Comparison local periodic, overlapping domains, and full wave calculations. Proceedings of the High Contrast Metastructures XIII.

[5] Chen W.T., Zhu A.Y., Sanjeev V., Khorasaninejad M., Shi Z., Lee E., Capasso F. (2018). A broadband achromatic metalens for focusing and imaging in the visible. Nat. Nanotechnol..

[6] Whiting E.B., Campbell S.D., Kang L., Werner D.H. (2020). Meta-atom library generation via an efficient multi-objective shape optimization method. Opt. Express.

[7] Zhang S., Khan K., Hellmann C., Wyrowski F. Modeling of diffractive-/metasurfaces and their performance evaluations in general optical systems. Proceedings of the Optical Modeling and Performance Predictions XI.

[8] Zhang S., Hellmann C., Wyrowski F. (2020). Modeling of high-contrast metasurfaces and their performance in general optical system using fast physical optics (Conference Presentation). Proceedings of the High Contrast Metastructures IX.

[9] Cai H., Srinivasan S., Czaplewski D.A., Martinson A.B.F., Gosztola D.J., Stan L., Loeffler T., Sankaranarayanan S.K.R.S., López D. (2020). Inverse design of metasurfaces with non-local interactions. NPJ Comput. Mater..

[10] Taflove A., Oskooi A.F., Johnson S.G. (2013). Advances in FDTD Computational Electrodynamics: Photonics and Nanotechnology.

[11] Lin Z., Johnson S.G. (2019). Overlapping domains for topology optimization of large-area metasurfaces. Opt. Express.

[12] Fernandez-Mouron L.-H., Tremas l., Legrand M., Urard P., Mohamad H., Dilhan L., Giuseppe Carnemolla E., Fissore M., Downing J., Serradeil V. Impact of Numerical Simulation Boundary-Decomposition Errors on Optical Performance of Metadiffusors. Proceedings of the The 14th International Conference on Metamaterials, Photonic Crystals and Plasmonics, META.

[13] Edee K., Granet G., Paladian F., Bonnet P., Al Achkar G., Damaj L., Plumey J.P., Larciprete M.C., Guizal B. (2022). Domain Decomposition Spectral Method Applied to Modal Method: Direct and Inverse Spectral Transforms. Sensors.

[14] Gao H.W., Xin X.M., Lim Q.J., Wang S., Peng Z. (2024). Efficient Full-Wave Simulation of Large-Scale Metasurfaces and Metamaterials. IEEE Trans. Antennas Propag..

[15] Phan T., Sell D., Wang E.W., Doshay S., Edee K., Yang J., Fan J.A. (2019). High-efficiency, large-area, topology-optimized metasurfaces. Light Sci. Appl..

[16] Linss S., Michaelis D., Zeitner U.D. (2021). Macroscopic wave-optical simulation of dielectric metasurfaces. Opt. Express.

[17] Torfeh M., Arbabi A. (2020). Modeling Metasurfaces Using Discrete-Space Impulse Response Technique. ACS Photonics.

[18] Gigli C., Li Q., Chavel P., Leo G., Brongersma M.L., Lalanne P. (2021). Fundamental Limitations of Huygens’ Metasurfaces for Optical Beam Shaping. Laser Photonics Rev..

[19] Bayati E. (2022). Design and Characterization of Optical Metasurface Systems. Ph.D. Thesis.

[20] Li L. (1993). Multilayer modal method for diffraction gratings of arbitrary profile, depth, and permittivity. J. Opt. Soc. Am. A.

[21] Li L. (1996). Formulation and comparison of two recursive matrix algorithms for modeling layered diffraction gratings. J. Opt. Soc. Am. A—Opt. Image Sci. Vis..

[22] Pérez-Arancibia C., Pestourie R., Johnson S.G. (2018). Sideways adiabaticity: Beyond ray optics for slowly varying metasurfaces. Opt. Express.

[23] Asoubar D., Zhang S., Wyrowski F., Kuhn M. (2012). Parabasal field decomposition and its application to non-paraxial propagation. Opt. Express.

[24] Li L. (2003). Note on the S-matrix propagation algorithm. J. Opt. Soc. Am. A.

[25] Wyrowski F., Kuhn M. (2011). Introduction to field tracing. J. Mod. Opt..

[26] Wyrowski F., Bryngdahl O. (1988). Iterative Fourier-transform algorithm applied to computer holography. J. Opt. Soc. Am. A.

[27] Hammond A.M., Oskooi A., Chen M., Lin Z., Johnson S.G., Ralph S.E. (2022). High-performance hybrid time/frequency-domain topology optimization for large-scale photonics inverse design. Opt. Express.

[28] Christiansen R.E., Lin Z., Roques-Carmes C., Salamin Y., Kooi S.E., Joannopoulos J.D., Soljačić M., Johnson S.G. (2020). Fullwave Maxwell inverse design of axisymmetric, tunable, and multi-scale multi-wavelength metalenses. Opt. Express.

